# Clinicopathological characteristics and survival outcomes in Paget disease: a SEER population‐based study

**DOI:** 10.1002/cam4.1475

**Published:** 2018-05-02

**Authors:** Yang Zhao, He‐Fen Sun, Meng‐Ting Chen, Shui‐Ping Gao, Liang‐Dong Li, Hong‐lin Jiang, Wei Jin

**Affiliations:** ^1^ Department of Breast Surgery Key Laboratory of Breast Cancer in Shanghai Collaborative Innovation Center of Cancer Medicine Fudan University Shanghai Cancer Center Shanghai 200030 China; ^2^ Department of Oncology Shanghai Medical College Fudan University Shanghai 200030 China; ^3^ Division of Molecular Medicine & Genetic Department of Internal Medicine and Life Sciences Institute University of Michigan Ann Arbor Michigan 48109

**Keywords:** Infiltrating ductal carcinoma, Paget disease, surveillance, epidemiology, and end results

## Abstract

The objective of this study was to investigate the clinicopathological characteristics and survival outcomes of Paget disease (PD), Paget disease concomitant infiltrating duct carcinoma (PD‐IDC), and Paget disease concomitant intraductal carcinoma (PD‐DCIS). We identified 501,631 female patients from 2000 to 2013 in the Surveillance, Epidemiology, and End Results (SEER) database. These identified patients included patients with PD (*n* = 469), patients with PD‐IDC (*n* = 1832), and patients with PD‐DCIS (*n* = 1130) and infiltrating ductal carcinoma (IDC) (*n* = 498,076). Then, we compared the clinical characteristics of these patients with those who were diagnosed with IDC during the same period. The outcomes of these subtypes of breast carcinoma were different. Based on the overall survival, the patients with PD‐IDC had the worst prognosis (5‐year survival rate = 84.1%). The PD‐DCIS had the best prognosis (5‐year survival rate = 97.5%). Besides, among patients with Paget disease, the one who was married had a better prognosis than who were not. And, according to our research, the marital status was associated with the hormone receptor status in patients with PD‐IDC. Among three subtypes of Paget disease, patients with PD‐IDC had the worst prognosis. Besides, patients who were unmarried had worse outcomes. And the marital status of patients with PD‐IDC is associated with hormone status. The observation underscores the importance of individualized treatment.

## Introduction

Breast cancer is the most common cancer in women across the world. According to the WHO experts in the world each year, there are revealed from 800,000 up to 1 million new cases of breast cancer 1. Paget disease is a rare form of breast cancer that occurs in the mouth of the excretory ducts of the nipple. This rare abnormality occurs in 0.5–5% of all cases of breast cancer 2. PD is characterized by an ulcerated, ulcerated, crusted, or scaling lesion on the nipple that can extend to the areola 3. Paget's disease of the nipple is characterized by histopathological infiltration of neoplastic cells with glandular features in the epidermal layer of the nipple–areolar complex. The pathologic mechanism of PD is still unclear. However, there are two kinds of explanation of the pathologic origin of the Paget disease epidermotropic and transformation theory 4, 5. The former one considered that the cells came from the underlying ductal tumor and then move along the lactiferous ducts to the nipple. And the other theory suggested that the cells were in situ in the major lactiferous sinuses.

Characterized by malignant crusting or ulceration of the nipple, Paget disease can present in one of three ways. The first one is in conjunction with an underlying invasive cancer. The second one is in conjunction with underlying ductal carcinoma in situ (DCIS). The last one is alone without any underlying invasive breast carcinoma or DCIS 6. The Paget disease can be treated by central lumpectomy with breast conservation. However, the prognosis of the PD is not well. IDC is the most common breast carcinoma subtype during the world. Recent study has suggested that patients with Paget disease conjunction with invasive cancer had worse prognosis 7. Nevertheless, study about all these three kinds of PD is not being researched. And study on relationship between PD and the IDC is rare. Previous study described that Paget disease alone without an underlying cancer is rare, and it presents utmost 8% of patients with Paget disease 8.

Married persons enjoy overall better health and increase life expectancy compared the unmarried (divorced, separated, and never married) 9, 10. Previous studies have indicated a survival advantage for married persons living with cancer 11, 12, 13. And a research found that married men and women with cancer to have a 15% reduced risk of death 14. We compared with unmarried men and women in different subtypes of Paget disease. Besides the different outcomes in unmarried patients, we found the correlation between the marital status and the hormone status and the human epidermal growth factor receptor II, which can guide the individualized treatment in clinic.

## Materials and Methods

### Ethics statement

We obtained permission to access the SEER research data. The data downloaded from the SEER do not require informed patient consent. Besides, our research was approved by the Ethical Committee and Institutional Review of Fudan University Shanghai Cancer Center (FDUSCC). The methods were performed in accordance with the approved guidelines.

### Data source

We examined the data from the National Cancer Institute's Surveillance, Epidemiology, and End Results (SEER) program, which contains the population‐based central cancer registries of 18 geographically defined regions. For this study, we use the November 2014–18 submission.

### Patient selection

We use the histopathology codes from the International Classification of Disease for Oncology third edition (ICD‐O‐3) to select female patients. In the ICD‐O‐3, the codes are defined as follows: code 8500 (ductal carcinoma), code 8540 (mammary Paget disease), code 8541 (Paget disease with infiltrating ductal carcinoma), and code 8543 (Paget disease with intraductal carcinoma). According to the ICD‐O‐3, we defined and choose the patients who had the PD (ICD‐O‐3 code 8540/3), PD‐IDC (ICD‐O‐3 code 85413), PD‐DCIS (ICD‐O‐3 code 8543/3), and IDC (ICD‐O‐3 code 8500/3). In this study, women who were diagnosed as all three kinds of PD and ICD between 2000 and 2013 were included (*n* = 501,631). And these identified patients included patients with PD (*n* = 469), patients with PD‐IDC (*n* = 1832), and patients with PD‐DCIS (*n* = 1130) and infiltrating ductal carcinoma (IDC) (*n* = 498,076).

### Statistical analysis

Overall survival (OS) was measured from the date on which the first‐time definite diagnosis was made until the date of death, the date last known to be alive, or September 2013. Disease‐specific survival (DSS) was measured from the date of diagnosis to the date of death which is associated with breast carcinoma. The National Cancer Institute's SEER*Stat software package (version 6.1.4; built on April 13, 2005) was used to calculate incidence rates. Baseline patient demographic characteristics and tumor information were compared using the Pearson's chi‐square test for categorical variables. Survival curves were plotted according to the Kaplan–Meier method and compared using the log‐rank test in a univariate analysis. Cox regression analysis was performed to compute hazard ratios and 95% confidence intervals (95% CIs) and to evaluate the effects of confounding factors. All the tests were two sided, and *P* values less than 0.05 were considered statistically significant. All the statistical analyses were performed using SPSS statistical software, version 22.0 (IBM Corp, Armonk, NY).

## Results

### Clinicopathological characteristics of PD

Overall 447,401 patients who were diagnosed with breast carcinoma were evaluated. We evaluated 447,401 patients with breast cancer. Among these patients, 443,970 were with infiltrating ductal breast carcinoma, 469 were with mammary Paget disease, 1832 were with Paget disease with infiltrating ductal carcinoma, and 1130 were with Paget disease with intraductal carcinoma. The demographics and clinicopathological characteristics of PD, PD‐IDC, and PD‐DCIS were compared with IDC. And the results are summarized in Table 1. Using the Pearson's chi‐square test, for PD and IDC, the significant variables were age (*P* < 0.001), marital status (*P* < 0.001), laterality (*P* < 0.001), tumor size (*P* < 0.001), lymph node status (*P* < 0.001), Grade (*P* < 0.001), AJCC stage (*P* < 0.001), ER (estrogen receptor) status (*P* < 0.001), PR (progesterone receptor) status (*P* < 0.001), HER2 (human epidermal growth factor receptor 2) status (*P* < 0.001), and whether had radiation treatment (*P* < 0.001). For PD‐IDC and IDC, the significant characteristics were race (*P* = 0.011), marital status (*P* < 0.001), tumor size (*P* < 0.001), lymph node status (*P* < 0.001), Grade (*P* < 0.001), AJCC stage (*P* < 0.001), ER status (*P* < 0.001), PR status (*P* < 0.001), HER2 status (*P* < 0.001), and whether had radiation treatment (*P* < 0.001). For PD‐DCIS and IDC, the considerable characteristics were age (*P* < 0.001), marital status (*P* < 0.001), tumor size (*P* < 0.001), Grade (*P* < 0.001), AJCC stage (*P* < 0.001), ER status (*P* < 0.001), PR status (*P* < 0.001), HER2 status (*P* < 0.001), and whether had radiation treatment (*P* < 0.001).

**Table 1:** Characteristics of patients with Paget disease and infiltrating duct carcinoma

Clinical characteristics		PD*N*	IDC*N*	*P*‐value	PD‐IDC*N*	IDC*N*	*P*‐value	PD‐DCIS*N*	IDC*N*	*P*‐value
Age at diagnosis (years)	18–49	114	158,076	<0.001	665	158,076	0.536	292	159,076	<0.001
	50–79	355	285,894		1167	285,894		838	285,894	
Race	White	393	360,769	0.111	1446	360,769	0.011	948	360,769	0.069
	Black	45	41,277		206	41,277		87	41,277	
	Other	31	41,924		180	41,924		95	41,924	
Marital status	Married	216	243,680	<0.001	903	243,680	<0.001	561	243,680	<0.001
	Not married	204	181,155		856	181,155		529	181,155	
	Unknown	49	19,134		73	19,134		40	19,134	
Laterality	Left	237	224,866	<0.001	959	224,866	0.446	614	224,866	0.066
	Right	226	218,611		872	218,611		516	218,611	
	Paired site	6	409		1	409		0	409	
	Unknown	0	84		0	84		0	84	
Tumor size (cm)	<2	54	25,463	<0.001	41	25,463	<0.001	20	25,463	<0.001
	2.1–5	249	280,120		1098	280,120		672	280,120	
	>5	9	7136		28	7136		6	7136	
	Unknown	157	131,251		665	131,251		432	131,251	
Lymph node status	Negative	158	257,428	<0.001	807	287,428	<0.001	645	257,428	0.539
	Positive	311	186,542		1025	186542		485	186,542	
Grade	I	11	84,295	<0.001	113	84,295	<0.001	17	84,295	<0.001
	II	23	176,027		526	176,027		108	176,027	
	III	41	160,309		1003	160,309		396	160,309	
	IV	3	5015		44	5015		237	5015	
	Unknown	391	18,324		146	18,324		372	18,324	
AJCC stage	0	83	5	<0.001	4	5	<0.001	160	5	<0.001
	I	11	70,594		153	70,594		19	70,594	
	II	2	42,900		106	42,900		11	42,900	
	III	4	13,995		95	13,995		3	13,995	
	IV	3	6346		21	6346		1	6346	
	Unknown	366	310,130		1453	310,130		936	310,130	
ER status	Negative	74	92,846	<0.001	769	92,846	<0.001	408	92,846	<0.001
	Positive	67	318,298		849	318,298		237	318,298	
	Borderline	0	701		11	701		1	701	
	Unknown	328	32,125		203	32,125		484	32,125	
PR status	Negative	95	136,827	<0.001	983	136,827	<0.001	467	136,827	<0.001
	Positive	37	268,719		613	268,719		138	268,719	
	Borderline	0	2063		11	2063		2	2063	
	Unknown	337	36,361		225	36,361		523	36,361	
HER2 status	Negative	7	106,696	<0.001	123	106,696	<0.001	7	106,696	<0.001
	Positive	17	21,261		210	21,261		33	21,261	
	Borderline	0	3124		8	3124		3	3124	
	Unknown	445	312,889		1491	312,889		1087	312,889	
Radiation	No	384	215,199	<0.001	1348	215,199	<0.001	918	215,199	<0.001
	Yes	67	213,217		435	213,217		191	213,217	
	Unknown	18	15,554		49	15,554		21	15,554	

AJCC, American Joint Committee on Cancer; ER, estrogen receptor; HER2, human epidermal growth factor receptor 2; IDC, infiltrating duct carcinoma; PD‐IDC, Paget disease concomitant infiltrating duct carcinoma; PD‐DCIS, Paget disease concomitant intraductal carcinoma, unmarried group included divorced, separated, single (never married), and widowed.

Table 2 presents the distribution of characteristics of women with breast cancer stratified by marital status. For patients with PD, the clinicopathologic characteristics were age at diagnosis (*P* = 0.002), race (*P* = 0.027), laterality (*P* = 0.004), tumor size (*P* < 0.001), lymph node status (*P* = 0.001) and radiation situation (*P* < 0.001). The hormone status did not have statistical significance. However, according to the analyses, patients who were diagnosed with PD‐IDC had different statistical factors. The hormone status had statistical significance—ER status (*P* = 0.01), PR status (*P* = 0.006), and HER2 status (*P* = 0.025). Meanwhile, for patients with PD‐DCIS, the associations were different again. Among the three hormones, only HER2 had statistical significance (*P* = 0.01). Other characteristics were age (*P* < 0.001), race (*P* = 0.012), and AJCC stage (*P* < 0.001). Be differ from the other two subtypes, the marital status of patients with PD‐DCIS had no significant correction with the radiation status.

**Table 2:** The association between clinical characteristics of Paget disease and marital status

Categories		Married (*n*)	Unmarried (*n*)	Unknown (*n*)	*P*‐value
				PD	
Age at diagnosis (years)	18–49	69	36	9	0.002
	50–79	147	168	40	
Race	White	187	170	36	0.027
	Black	16	24	5	
	Other	13	10	8	
Laterality	Left	109	113	15	0.004
	Right	107	86	33	
	Paired site	0	5	1	
	Unknown	0	0	0	
Tumor size (cm)	<2	16	25	13	<0.001
	2.1–5	134	101	14	
	>5	0	5	4	
	Unknown	66	73	18	
Lymph node status	Negative	84	69	5	0.001
	Positive	132	135	44	
Grade	I	6	5	0	0.523
	II	10	13	0	
	III	22	14	5	
	IV	2	1	0	
	Unknown	176	171	44	
AJCC stage	0	49	28	6	0.177
	I	7	3	1	
	II	0	1	1	
	III	2	2	0	
	IV	2	1	0	
	Unknown	156	169	41	
ER status	Negative	33	34	7	0.249
	Positive	38	26	3	
	Borderline	145	144	39	
	Unknown	216	204	49	
PR status	Negative	48	39	8	0.641
	Positive	18	17	2	
	Borderline	0	0	0	
	Unknown	150	148	39	
HER2 status	Negative	4	3	0	0.695
	Positive	10	6	1	
	Borderline	0	0	0	
	Unknown	202	195	48	
Radiation	No	174	173	37	<0.001
	Yes	36	27	4	
	Unknown	6	4	8	
		PD‐IDC			
Age at diagnosis (years)	18–49	407	240	18	<0.001
	50–79	496	616	55	
Race	White	740	653	53	<0.001
	Black	58	137	11	
	Other	105	66	9	
Laterality	Left	481	443	35	0.715
	Right	422	412	38	
	Paired site	0	1	0	
	Unknown	0	0	0	
Tumor size (cm)	<2	14	23	4	0.189
	2.1–5	553	506	39	
	>5	14	14	0	
	Unknown	322	313	30	
Lymph node status	Negative	407	366	34	0.562
	Positive	496	490	39	
Grade	I	43	67	3	0.169
	II	266	236	24	
	III	498	469	36	
	IV	25	18	1	
	Unknown	71	66	9	
AJCC stage	0	1	3	0	0.411
	I	86	60	7	
	II	45	58	3	
	III	49	41	5	
	IV	13	8	0	
	Unknown	709	686	58	
ER status	Negative	397	347	25	0.01
	Positive	424	391	34	
	Borderline	3	8	0	
	Unknown	79	110	14	
PR status	Negative	492	456	35	0.006
	Positive	314	279	20	
	Borderline	5	4	2	
	Unknown	92	117	16	
HER2 status	Negative	56	63	4	0.025
	Positive	114	88	8	
	Borderline	5	1	2	
	Unknown	728	704	59	
Radiation	No	634	660	45	<0.001
	Yes	244	174	17	
	Unknown	16	22	11	
Age at diagnosis (years)	18–49	407	240	18	<0.001
	50–79	496	616	55	
Race	White	740	653	53	<0.001
	Black	58	137	11	
	Other	105	66	9	
Laterality	Left	481	443	35	0.715
	Right	422	412	38	
	Paired site	0	1	0	
	Unknown	0	0	0	
Tumor size (cm)	<2	14	23	4	0.189
	2.1–5	553	506	39	
	>5	14	14	0	
	Unknown	322	313	30	
Lymph node status	Negative	407	366	34	0.562
	Positive	496	490	39	
Grade	I	43	67	3	0.169
	II	266	236	24	
	III	498	469	36	
	IV	25	18	1	
	Unknown	71	66	9	
AJCC stage	0	1	3	0	0.411
	I	86	60	7	
	II	45	58	3	
	III	49	41	5	
	IV	13	8	0	
	Unknown	709	686	58	
ER status	Negative	397	347	25	0.01
	Positive	424	391	34	
	Borderline	3	8	0	
	Unknown	79	110	14	
PR status	Negative	492	456	35	0.006
	Positive	314	279	20	
	Borderline	5	4	2	
	Unknown	92	117	16	
HER2 status	Negative	56	63	4	0.025
	Positive	114	88	8	
	Borderline	5	1	2	
	Unknown	728	704	59	
Radiation	No	634	660	45	<0.001
	Yes	244	174	17	
	Unknown	16	22	11	

AJCC, American Joint Committee on Cancer; ER, estrogen receptor; HER2, human epidermal growth factor receptor 2; IDC, infiltrating duct carcinoma; PD‐IDC, Paget disease concomitant infiltrating duct carcinoma; PD‐DCIS, Paget disease concomitant intraductal carcinoma, unmarried group included divorced, separated, single (never married), and widowed.

### Comparison of survival between three subtypes of Paget disease and IDC

**Figure 1:**
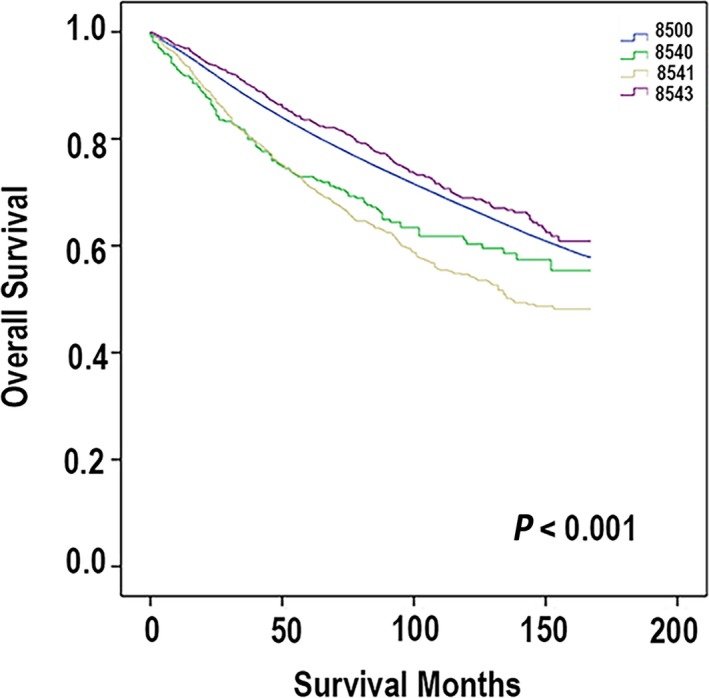
According to the ICD‐O‐3, the codes are defined: code 8500 (ductal carcinoma), code 8540 (mammary Paget disease), code 8541 (Paget disease with infiltrating ductal carcinoma), and code 8543 (Paget disease with intraductal carcinoma). Overall survival (OS) was measured from the date on which the first‐time definite diagnosis was made until the date of death, the date last known to be alive, or September 2013.

**Figure 2:**
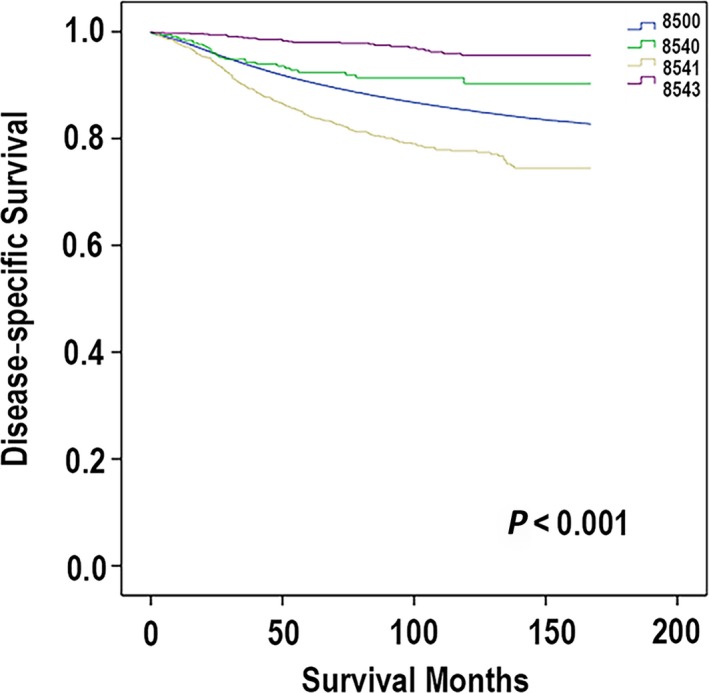
According to the ICD‐O‐3, the codes are defined: code 8500 (ductal carcinoma), code 8540 (mammary Paget disease), code 8541 (Paget disease with infiltrating ductal carcinoma), and code 8543 (Paget disease with intraductal carcinoma). Disease‐specific survival (DSS) was measured from the date of diagnosis to the date of death which is associated with breast carcinoma.

Utilizing the Kaplan–Meier method, we analyzed all these four subtypes (PD, PD‐IDC, PD‐DCIS, and IDC) of mammary carcinoma. On the basis of the OS, the different outcomes of four subtypes of breast carcinoma are shown distinctly in Figure 1. Patients with PD‐DCIS had the best prognosis with a 5‐year OS 83.6%. The one worse than the PD‐DCIS was IDC. The 5‐year OS of patients with IDC was 81.1%. Then, the next one was PD. The 5‐year OS of patients with PD was 72.9%. The one with worst outcomes was PD‐IDC, whose 5‐year OS was 71.4%. Then, we analyzed the cases utilizing the DSS, and the comparison of different kinds of mammary cancer is shown in Figure 2. The patients with PD‐DCIS had the best prognosis with a 5‐year survival rate of 98.2%. The worse one was patients with PD. Its 5‐year survival rate was 92.4%. The survival rate of patients with IDC was 91%. And patients who were diagnosed with PD‐IDC had the worst outcomes. Its 5‐year survival rate was 84.1%. Apparently, the results of the analyses based on the OS and DSS had a little difference. Based on the OS, the results showed that the prognosis of PD was worse than IDC. However, based on the DSS, the outcome of the IDC was worse than PD. Meanwhile, the prognostic indicators can be found during the univariate analysis.

### The survival analyses in subtypes of Paget disease

According to the Kaplan–Meier method and compared utilizing the log‐rank test, we analyzed the Paget disease and its indicator which were associated with the prognosis. The results of the analyses are shown in Table 3. For PD, indicators which had significance were age at diagnosis (*P* < 0.001), marital status (*P* < 0.001), tumor size (*P* < 0.001), lymph node status (*P* < 0.001), and AJCC stage (*P* < 0.001). For PD‐IDC, the significant indicators were age at diagnosis, marital status, tumor size, lymph node status, Grade, AJCC stage, and ER status. Meanwhile, the significant indicators of PD‐DCIS were age at diagnosis (*P* < 0.001), marital status (*P* < 0.001), tumor size (*P* < 0.001), lymph node status (*P* < 0.001), AJCC stage (*P* < 0.001), HER2 status (*P* < 0.001), and radiation or not (*P* = 0.007).

**Table 3:** Survival analyses–univariate analyses of Paget disease

PD			PD‐IDC			PD‐DCIS		
Variables	Category	*P*‐value	Variables	Category	*P*‐value	Variables	Category	*P*‐value
Age at diagnosis (years)	18–49	<0.001	Age at diagnosis (years)	18–49	<0.001	Age at diagnosis (years)	18–49	<0.001
	50–79			50–79			50–79	
Race	White	0.052	Race	White	0.296	Race	White	0.253
	Black			Black			Black	
	Other			Other			Other	
Marital status	Married	<0.001	Marital status	Married	<0.001	Marital status	Married	<0.001
	Not married			Not married			Not married	
	Unknown			Unknown			Unknown	
Laterality	Left	0.112	Laterality	Left	0.561	Laterality	Left	0.162
	Right			Right			Right	
	Paired site			Paired site			Paired site	
	Unknown			Unknown			Unknown	
Tumor size (cm)	<2	<0.001	Tumor size (cm)	<2	<0.001	Tumor size (cm)	<2	<0.001
	2.1–5			2.1–5			2.1–5	
	>5			>5			>5	
	Unknown			Unknown			Unknown	
Lymph node status	Negative	<0.001	Lymph node status	Negative	<0.001	Lymph node status	Negative	<0.001
	Positive			Positive			Positive	
Grade	I	0.069	Grade	I	0.016	Grade	I	0.313
	II			II			II	
	III			III			III	
	IV			IV			IV	
	Unknown			Unknown			Unknown	
AJCC stage	0	<0.001	AJCC stage	0	<0.001	AJCC stage	0	<0.001
	I			I			I	
	II			II			II	
	III			III			III	
	IV			IV			IV	
	Unknown			Unknown			Unknown	
ER status	Negative	0.954	ER status	Negative	0.004	ER status	Negative	0.363
	Positive			Positive			Positive	
	Borderline			Borderline			Borderline	
	Unknown			Unknown			Unknown	
PR status	Negative	0.758	PR status	Negative	0.055	PR status	Negative	0.565
	Positive			Positive			Positive	
	Borderline			Borderline			Borderline	
	Unknown			Unknown			Unknown	
HER2 status	Negative	0.161	HER2 status	Negative	0.348	HER2 status	Negative	<0.001
	Positive			Positive			Positive	
	Borderline			Borderline			Borderline	
	Unknown			Unknown			Unknown	
Radiation	No	0.085	Radiation	No	0.077	Radiation	No	0.007
	Yes			Yes			Yes	
	Unknown			Unknown			Unknown	

AJCC, American Joint Committee on Cancer; ER, estrogen receptor; HER2, human epidermal growth factor receptor 2; IDC, infiltrating duct carcinoma; PD‐IDC, Paget disease concomitant infiltrating duct carcinoma; PD‐DCIS, Paget disease concomitant intraductal carcinoma, unmarried group included divorced, separated, single (never married), and widowed.

Using Cox regression analysis was performed to compute hazard ratios and 95% confidence intervals. Choosing the variates which were significant in the univariate analyses, the multivariate analysis was performed. And the results are shown in Table 4. For PD, significant indicators of prognosis were age at diagnosis (*P* = 0.005, HR = 0.449, 95% CI, 0.257–0.787), race (*P* = 0.014), marital status (*P* < 0.001), tumor size (*P* = 0.033), lymph node status (*P* < 0.001, positive, HR = 0.417, 95% CI, 0.264–0.658), and Grade (*P* = 0.042). The *P*‐value of AJCC stage was larger than 0.05 (*P* = 0.203). For PD‐IDC, variates which had prognostic significance were age at diagnosis (*P* < 0.001, HR = 0.347, 95% CI, 0.283–0.425), marital status (*P* < 0.001), tumor size (*P* < 0.001), lymph node status (*P* < 0.001, positive, HR = 0.437, 95% CI, 0.366–0.522), Grade (*P* = 0.049), AJCC stage (*P* < 0.001), and ER status (*P* = 0.034, positive, HR = 0.453, 95% CI, 0.195–1.052). The statistic significant indicators of the patients with PD‐DCIS were age at diagnosis (*P* < 0.001, HR = 0.309, 95% CI, 0.203–0.469), marital status (*P* < 0.001, not married, HR = 0.504, 95% CI, 0.269–0.945), tumor size (*P* < 0.001), lymph node status (*P* < 0.001, positive, HR = 0.546, 95% CI, 0.424–0.704), HER2 status (*P* = 0.004, positive, HR = 9.502, 95% CI, 2.758–32.734), and radiation or not *P* = 0.001, yes, HR = 2.183, 95% CI, 0.688–6.922).

**Table 4:** Survival analyses–multivariate analyses of Paget disease

Variables	Category	Hazard ratio	95% Confidence interval	*P*‐value
PD				
Age at diagnosis (years)	18–49	1	Referent	0.005
	50–79	0.449	0.257–0.787	
Race	White	1	Referent	0.014
	Black	3.772	1.366–10.413	
	Other	5.495	1756–17.2	
Marital status	Married	1	Referent	<0.001
	Not married	0.379	0.214–0.672	
	Unknown	0.887	0.528–1.491	
Tumor size (cm)	<2	1	Referent	0.033
	2.1–5	1.417	0.806–2.494	
	>5	0.651	0.429–0.988	
	Unknown	1.506	0.509–4.454	
Lymph node status	Negative	1	Referent	<0.001
	Positive	0.417	0.264–0.658	
Grade	I	1	Referent	0.042
	II	1.065	0.3–2.86	
	III	2.537	1.239–5.139	
	IV	0.714	0.313–1.628	
	Unknown	1.404	0.189–10.436	
AJCC stage	0	1	Referent	0.203
	I	0.795	0.353–1.793	
	II	0	0	
	III	0	0	
	IV	1.613	0.204–12.763	
	Unknown	5.224	1.449–18.837	
PD‐IDC				
Age at diagnosis (years)	18–49	1	Referent	<0.001
	50–79	0.347	0.283–0.425	
Race	White	1	Referent	0.77
	Black	0.556	0.813–1.47	
	Other	0.472	0.795–1.643	
Marital status	Married	1	Referent	<0.001
	Not married	0.625	0.427–0.914	
	Unknown	1.053	0.728–1.523	
Tumor size (cm)	<2	1	Referent	<0.001
	2.1–5	2.537	1.662–3.873	
	>5	0.915	0.769–1.088	
	Unknown	1.255	0.685–2.302	
Lymph node status	Negative	1	Referent	<0.001
	Positive	0.437	0.366–0.522	
Grade	I	1	Referent	0.049
	II	0.696	0.439–1.103	
	III	0.946	0.683–1.311	
	IV	1.155	0.855–1.561	
	Unknown	0.855	0.705–2.256	
AJCC stage	0	1	Referent	<0.001
	I	0	0	
	II	0.548	0.256–1.172	
	III	0.67	0.329–1.364	
	IV	1.055	0.632–1.764	
	Unknown	4.754	2.48–9.112	
ER status	Negative	1	Referent	0.034
	Positive	0.453	0.195–1.052	
	Borderline	0.438	0.19–1.007	
	Unknown	1.329	0.373–4.732	
PR status	Negative	1	Referent	0.212
	Positive	2.12	0.931–4.827	
	Borderline	1.818	0.799–4.138	
	Unknown	2.477	0.66–9.29	
PD‐DCIS				
Age at diagnosis (years)	18–49	1	Referent	<0.001
	50–79	0.309	0.203–0.469	
Race	White	1	Referent	0.63
	Black	1.058	0.619–1.808	
	Other	1.288	0.67–2.475	
Marital status	Married	1	Referent	<0.001
	Not married	0.504	0.269–0.945	
	Unknown	1.237	0.675–2.266	
Tumor size (cm)	<2	1	Referent	<0.001
	2.1–5	4.82	2.351–9.88	
	>5	1.035	0.772–1.388	
	Unknown	1.617	0.218–11.983	
Lymph node status	Negative	1	Referent	<0.001
	Positive	0.546	0.424–0.704	
Grade	I	1	Referent	0.332
	II	0.35	0.085–1.447	
	III	0.74	0.457–1.198	
	IV	0.891	0.663–1.198	
	Unknown	0.786	0.569–1.088	
ER status	Negative	1	Referent	0.3
	Positive	1.424	0.759–2.672	
	Borderline	0.922	0.486–1.749	
	Unknown	0.968	0.23–14.54	
PR status	Negative	1	Referent	0.898
	Positive	0.857	0.467–1.574	
	Borderline	1.047	0.513–2.134	
	Unknown	0	0	
HER2 status	Negative	1	Referent	0.004
	Positive	9.502	2.758–32.734	
	Borderline	0.614	0.084–4.466	
	Unknown	0	0	
Radiation	No	1	Referent	0.001
	Yes	2.183	0.688–6.922	
	Unknown	1.096	0.33–3.638	

AJCC, American Joint Committee on Cancer; ER, estrogen receptor; HER2, human epidermal growth factor receptor 2; IDC, infiltrating duct carcinoma; PD‐IDC, Paget disease concomitant infiltrating duct carcinoma; PD‐DCIS, Paget disease concomitant intraductal carcinoma, unmarried group included divorced, separated, single (never married), and widowed.

### The association between Paget disease and patient's marital status

**Figure 3:**
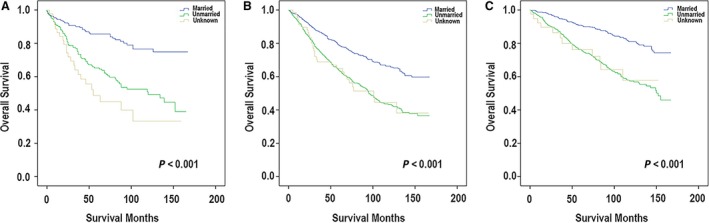
According to the Kaplan–Meier method and compared using the log‐rank test, we analyzed the Paget disease and the marital status. (A) The association between marital status and clinical prognosis in patients with PD. (B) The association between marital status and clinical prognosis in patients with PD‐IDC. (C) The association between marital status and clinical prognosis in patients with PD‐DCIS.

According to the Kaplan–Meier method and compared using the log‐rank test, we analyzed the Paget disease and the marital status. And Figure 3 presents the correlation. For patients with PD (Fig. 3A), the married patients had the best prognosis with a 5‐year OS of 85.6%. The unmarried patients (included single patients who never married, widowed, divorced, and separated patients) had worse outcomes with a 5‐year OS of 65.2%. Patients whose marital status was unknown had the worst diagnosis with a 5‐year OS of 48.7%. And the difference between them had statistical significance (*P* < 0.001). For patients who were diagnosed with PD‐IDC (Fig. 3B), the married patients had the best prognosis with a 5‐year OS of 78.7%. The next was patients who were unmarried with a 5‐year OS of 64.1%. For this subtype, the patients whose marital status was unknown had the almost similar 5‐year OS of 64.9%. And the difference was statistically significant as well (*P* < 0.001). For patients with PD‐DCIS (Fig. 3C), the 5‐year OS was 90.8% (married), 76.3% (unmarried), and 76.2% (unknown).

## Discussion

Previous study had reported that patients who were diagnosed of Paget disease with underlying invasive cancer had poor tumor characteristics 15. A previous research showed that the Paget disease with underlying invasive cancer had tumors with Grade 3 histology 8. In 1881, Thin observed that the nipple lesion contained malignant cells which were correlated to the underlying cancer 16. And this observation suggested the process of intraductal extension of cancer through the major lactiferous sinuses. We call it “pagetoid spread” nowadays. Histologically, Paget cells are large cells with pale, clear cytoplasm. It has enlarged nucleoli located within the epidermis and along the basal layer. The most widely accepted hypothesis to explain the origin of Paget cells is the epidermotropic theory. And this theory considered that Paget cells are derived from an underlying mammary adenocarcinoma 17. Evidence supporting the epidermotropic theory is based on studies showing that Paget disease is associated with an underlying breast carcinoma in most patients 18, 19, 20. Binding of heregulin to its receptor on Paget cells can induce chemotaxis of these breast cancer cells, and the cells eventually migrate into the overlying nipple epidermis 21. It is noteworthy that Paget cells and the underlying associated ductal carcinoma share the same immunohistochemical profile 22 and the same patterns of gene expression.

In allusion to different subtype of Paget disease, we found that the significantly associated indicators were different. Unmarried patients of PD, including those who were widowed, divorced, and never married, were at significantly great risk of existing lymph node metastasis. Meanwhile, for patients of PD‐IDC, we found that the hormone status was related to the human epidermal growth factor receptor II. However, for the patients with PD‐DCIS, only human epidermal growth factor receptor II had statistical significance. The association between marital status and these indicators was significant for every malignancy evaluated. Previous studies have linked marriage to improvements in cardiovascular, endocrine, and immune function, and marriage may be a determinant of the magnitude and presence of this effect 23, 24. Cortisol levels seem to be lower in patients with cancer who have adequate support networks, and diurnal cortisol patterns have been linked with natural killer cell count and survival in patients with cancer^25^, ^26^, potentially providing a physiologic basis for the psychologically based data described previously 27. Further investigations on this subject are warranted.

However, the study also had some limitations. The SEER database did not give us enough information about the lymphovascular invasion which can be regarded as the prediction of lymph node metastasis. Besides, the follow‐up of many patients was limited. And the information of systemic therapy of the patients was lack according to the SEER system. Based on the SEER database, the HER2 status was tested from 2010; however, the cases were from 2000 to 2013. Apparently, analyses of the HER2 were limited. And it made us unable to explore the clinical significance of HER2 status. Therefore, our study was limited by lack of some information. Besides, there is potential for misclassification of marital status. We did not take into account changes of marital status which may have occurred during the follow‐up period. And this phenomenon may have influenced our results. Thus, our findings may underestimate the protective effect that marriage has on breast cancer outcome. We defined that the single category contained divorcees, widows, and never married women. However, previous studies had found that there may be some difference among groups of unmarried women. Although the difference existed, the unmarried women fare worse than the married counterparts.

In conclusion, our study showed patients with PD‐IDC have the worst prognosis. Among all these three kinds of Paget disease, unmarried patients had worse outcomes. And the marital status of patients with PD‐IDC is associated with hormone status and HER2 status. The observation underscores the importance of individualized treatment.

## Conflict of Interests

The authors declare no conflict of interests.
